# A New Fern-like Plant *Xinhangia spina* Gen. et sp. Nov. from the Upper Devonian of China

**DOI:** 10.3390/biology11111568

**Published:** 2022-10-26

**Authors:** Jiang-Nan Yang, De-Ming Wang

**Affiliations:** Key Laboratory of Orogenic Belts and Crustal Evolution, Department of Geology, Peking University, Beijing 100871, China

**Keywords:** fern-like plants, triseriate branching, Late Devonian, stele, *Xinhangia*, Wutong Formation

## Abstract

**Simple Summary:**

In 2019, Late Devonian Xinhang forest was reported from Anhui Province, China. It represents the earliest forest in Asia and China, and was regarded as monospecific with numerous small trees of the lycopsid. Recently, we found some other plants from the forest and now erect a new fern-like plant named *Xinhangia*. It is small with height of tens of centimeters, and usually has two orders of branches in alternate and sometimes triseriate pattern. Spines densely occur on the main axes and primary branches. Its leaf-like organs are very simple and dichotomize into recurved tips. Fertile organs are similar to the “leaves” but terminated in elongate and paired sporangia. Both the “leaves” and fertile organs are alternately arranged on secondary branches. In anatomy, the primary xylem is “8”-shaped and is surrounded by secondary xylem. With simple characters, *Xinhangia* represents a morphologically primitive plant and is of uncertain affinity at class or order level. As a component of Xinhang forest, *Xinhangia* will help understand the growth habit and habitat of fern-like plants, as well as the structure and ecology of ancient forests in the future.

**Abstract:**

Palaeozoic fern-like plants show great diversity in their morphology and/or anatomy. Within this group, a novel taxon, *Xinhangia spina* gen. et sp. nov., is now reported from the Upper Devonian (Famennian) Wutong Formation of Anhui Province, China. The primary and secondary branches are borne alternately and sometimes in a triseriate pattern. Spines are evident on the main axes or stems and on the primary branches. Vegetative ultimate appendages with recurved tips are alternate, usually dichotomous 1–2 times, and sometimes as an aphlebia located at the base of primary or secondary branches. Fertile ultimate appendages are alternate, usually dichotomous 1–2 times, and terminate in elongated and paired sporangia. The stele has a clepsydroid-like primary xylem with each end bearing a protoxylem strand. The secondary xylem surrounding the primary xylem illustrates uniseriate rays. With rare divisions in both the vegetative and fertile ultimate appendages, *Xinhangia* represents a morphologically primitive plant. It is of uncertain affinity at the class or order level. The stelar architecture suggests that the clepsydroid stele may not be emphasized in discussing the relationship among fern-like plants such as rhacophytaleans.

## 1. Introduction

Ferns with megaphylls (laminate leaves containing multiple veins) bearing sporangia originated in the Carboniferous and have evolved until now, while fern-like plants (as probable fern precursors), without foliar-borne sporangia, usually existed in the Middle Devonian–Carboniferous [1,2,3,4,5]. The fern-like plants include the iridopteridaleans, pseudosporochnaleans, nonpseudosporochnaleans, rhacophytaleans and stauropteridaleans. Generally, they have sporangia-terminating branches, lack megaphylls and are anatomically characterized by a permanent protoxylem located near the periphery of mesarch primary xylem segments. In fern-like plants, the composition of rhacophytaleans (Rhacophytales) is controversial and the relationship among the members remains unclear [2,3,6].

Here, we report a new fern-like plant, *Xinhangia spina* gen. et sp. nov. from the Upper Devonian in China. On the basis of its fertile and vegetative morphology and associated anatomy, *Xinhangia* is compared with other fern-like plants and its affinity is discussed. The anatomical features of the rhacophytaleans are reconsidered.

## 2. Materials and Methods

Specimens were collected from the lowermost part of Leigutai Member (Wutong Formation), in the Yongchuan clay mine near Jianchuan village, Xinhang Town, Guangde City, Anhui Province, China (referring to Figure 1A in [7]). The Yongchuan mine is still under excavation and has a 60-hectare excavated area at present. Wang et al. [7] have briefly described the strata near Xinhang Town, which mainly includes the Leigutai Member and underlying Guanshan Member of the Upper Devonian Wutong Formation. The dominant species of the Xinhang Forest, arborescent *Guangdedendron micrum* [7,8], is widely distributed in the rock layers of the Leigutai Member at the Yongchuan mine. The stratum containing this new plant is ca. 4 m thick and mainly constituted of yellow or grey sandstone, siltstone and mudstone.

More than 100 specimens were collected for a detailed study. Steel needles were used to expose the morphology of the plant under a light microscope (LM). The permineralized axes were embedded, sectioned and ground to show the anatomy, and some of them were macerated with hydrofluoric acid (HF). The anatomy was observed under an LM through reflected light. Some tracheids were observed with a scanning electron microscope (SEM). By extensive observation under the microscope, no *in situ* spores were found from any of the sporangia. The photographs were taken with a digital camera and the LM. All the figures were prepared using Adobe Photoshop CC 2018 and Adobe Illustrator CC 2018 software. All the specimens illustrated in this paper are housed at the Department of Geology, Peking University, Beijing, China.

## 3. Systematics Palaeontology

**Figure 1:**
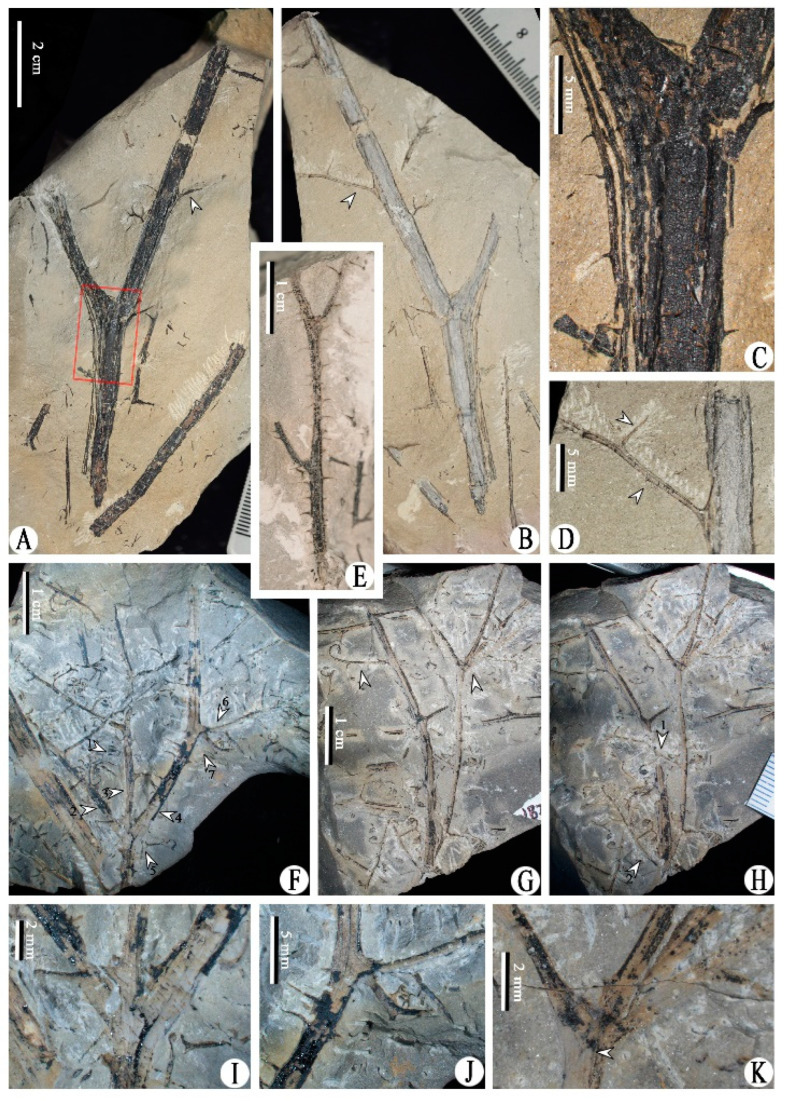
Morphology of *Xinhangia spina* gen. et sp. nov. from Guangde City, Anhui Province, China. (**A**,**B**) Part and counterpart of dichotomous main axis bearing one primary branch (arrow). (**C**) Enlargement of (**A**) (rectangle area) showing the spines on the main axis. (**D**) Enlargement of (**B**) (arrow) showing primary branch bearing two alternate secondary branches. (**E**) Main axis with dense spines and two alternate and spiny primary branches. (**F**) Main axis (arrow 4) with a pair (arrow 2, 3) and a single (arrow 6) primary branch in a triseriate pattern. Arrow 1 indicates single sporangial cluster enlarged in Figure 5A. (**G**,**H**) Two stages of dégagement showing two primary branches bearing fertile secondary branches alternately. (**I**) Enlargement of (**F**) (arrow 5) showing basal position of the pair of primary branches. (**J**) Enlargement of (**F**) (arrow 7) showing dichotomous aphlebia at base of primary branch. (**K**) Enlargement of (**G**) (right) showing attachment of paired and a single primary branch. Arrow indicates branch scar.

**Figure 2:**
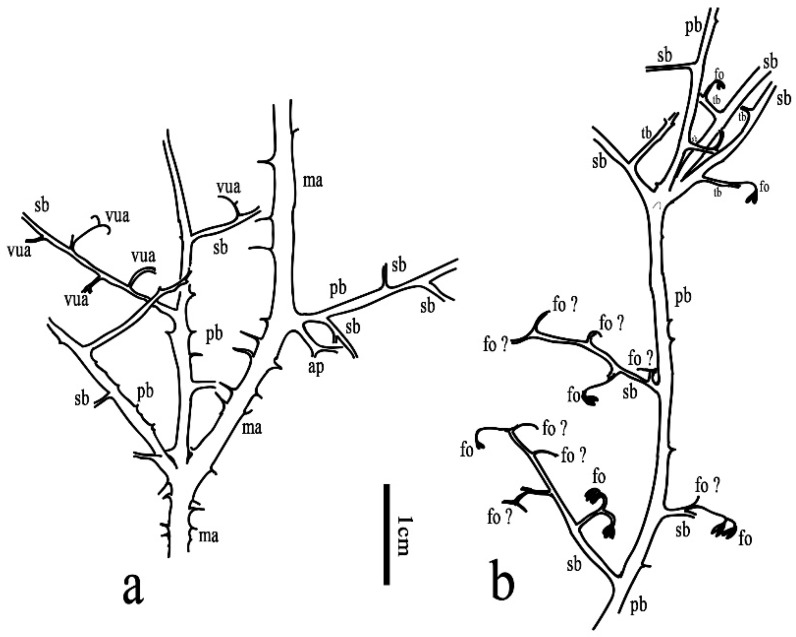
Morphology of *Xinhangia spina* gen. et sp. nov. from Guangde City, Anhui Province, China. (**a,b**) Line drawing of Figure 1F,H, respectively, ma, main axis; pb, primary branch; sb, secondary branch; tb, tertiary branch; vua, vegetative ultimate appendage; ap: aphlebia; fo, fertile organ; fo ?: assumed fertile organ.

**Figure 3:**
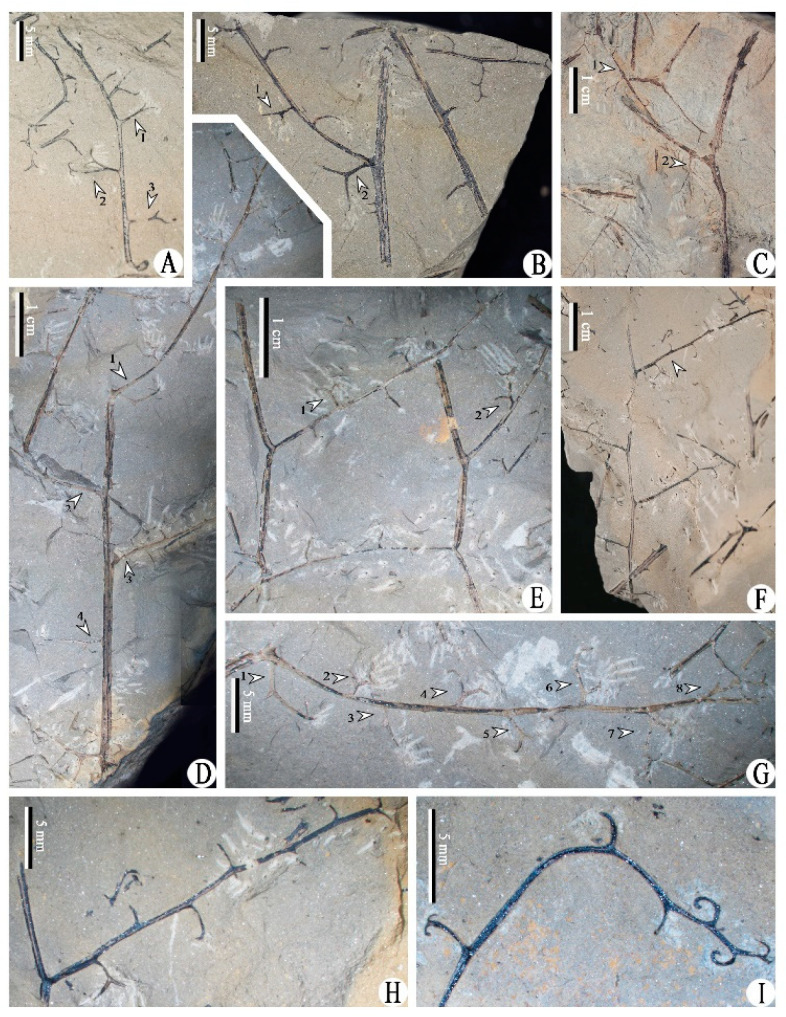
Morphology of *Xinhangia spina* gen. et sp. nov. from Guangde City, Anhui Province, China. (**A**) Secondary branch bearing vegetative ultimate appendages (arrows) in alternate arrangement. (**B**,**C**) Primary branch with secondary branch bearing vegetative ultimate appendages alternately. Arrow 1 in (**B**) indicates a dichotomized vegetative ultimate appendage. Arrow 2 in (**B**) and arrow 2 in (**C**) indicate aphlebia at the base of secondary branch. Arrow 1 in (**C**) indicates secondary branch. (**D**–**F**) Primary branch bearing secondary branches alternately. Arrows 1–4 in (**D**) and arrow in (**F**) indicate secondary branches. Arrows 1 and 2 in (**E**) indicate dichotomized vegetative ultimate appendages. (**G**) Enlargement of **D** (arrow 1) showing secondary branch bearing vegetative ultimate appendages alternately (arrows 2–8) and one aphlebia at base (arrow 1). (**H**) Enlargement of (**F**) (arrow) showing secondary branch bearing vegetative ultimate appendages alternately. (**I**) Upper portion of secondary branch bearing vegetative ultimate appendages alternately.

**Figure 4:**
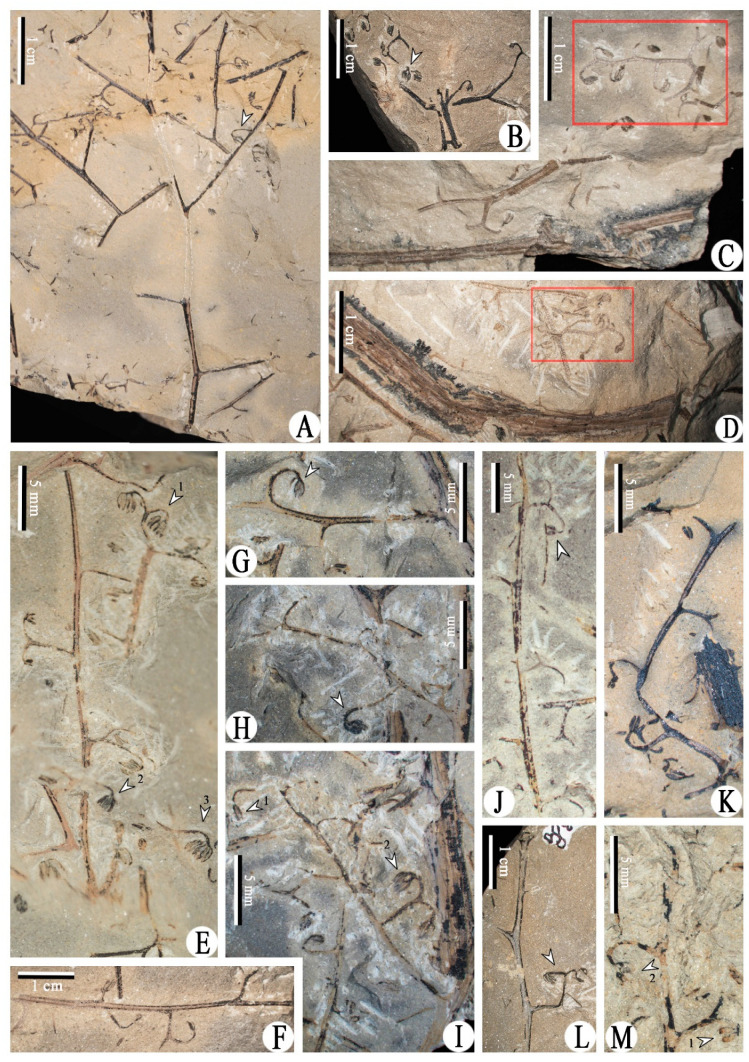
Morphology of *Xinhangia spina* gen. et sp. nov. from Guangde City, Anhui Province, China. (**A**) Fertile primary branch alternately bearing four secondary branches with one fertile organ connected (arrow). (**B**) Fertile secondary branches bearing fertile organs. Dispersed fertile organs in matrix. (**C**,**D**) Relatively complete fertile secondary branch near a main axis, bearing fertile organs alternately. (**E**–**M**) Secondary branches bearing fertile organs alternately. (**G**–**I**) represent enlargement from Figure 1G (left arrow) and 1H (arrows 1, 2), respectively. All the sporangia indicated by arrows are enlarged in Figure 5.

**Figure 5:**
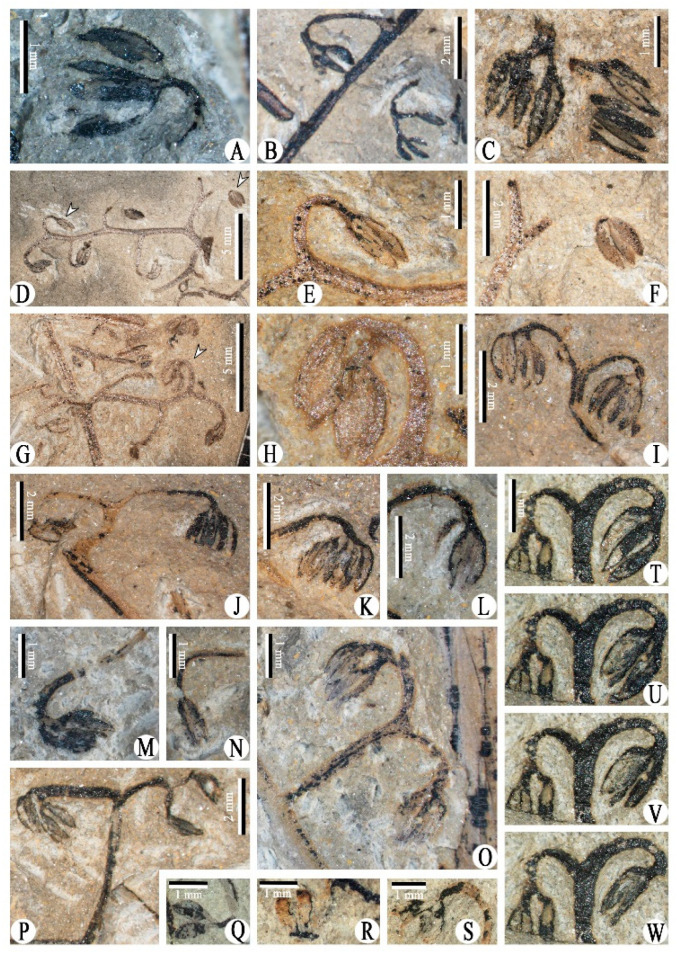
Morphology of *Xinhangia spina* gen. et sp. nov. from Guangde City, Anhui Province, China. (**A**) One single sporangial cluster bearing two pairs of elongate sporangia enlarged from Figure 1F (arrow 1). (**B**) Enlargement of Figure 4A (arrow) showing one fertile organ arranged on secondary branch. (**C**) Two clusters of sporangia enlarged from Figure 4B (arrow). (**D**,**G**) Fertile secondary branches bearing fertile organs enlarged from the rectangle areas in Figure 4C and 4D, respectively. (**E**) Enlargement of (**D**) (left arrow) showing one fertile organ with one pair of sporangia. (**F**) Enlargement of (**D**) (right arrow) showing dichotomous fertile organ with terminal sporangial cluster. (**H**) Enlargement of (**G**) (arrow) showing one fertile organ consisting of two pairs of sporangia. (**I**,**J**,**O**,**P**) Dichotomous fertile organ consisting of two major clusters bearing two to four pairs of sporangia. Enlarged from Figure 4E (arrows 1, 2), 4I (arrow 2), 4L (arrow), respectively. (**K**–**N**,**Q**–**S**) Single cluster of sporangia consisting of one to four pairs. Enlarged from Figure 4E (arrow 3), 4G (arrow), 4H (arrow), 4I (arrow 1), 4J (arrow), 4M (arrows 1, 2), respectively. (**T**–**W**) Serial dégagement of one cluster of fertile organs showing that the cluster consists of four sporangia.

**Figure 6:**
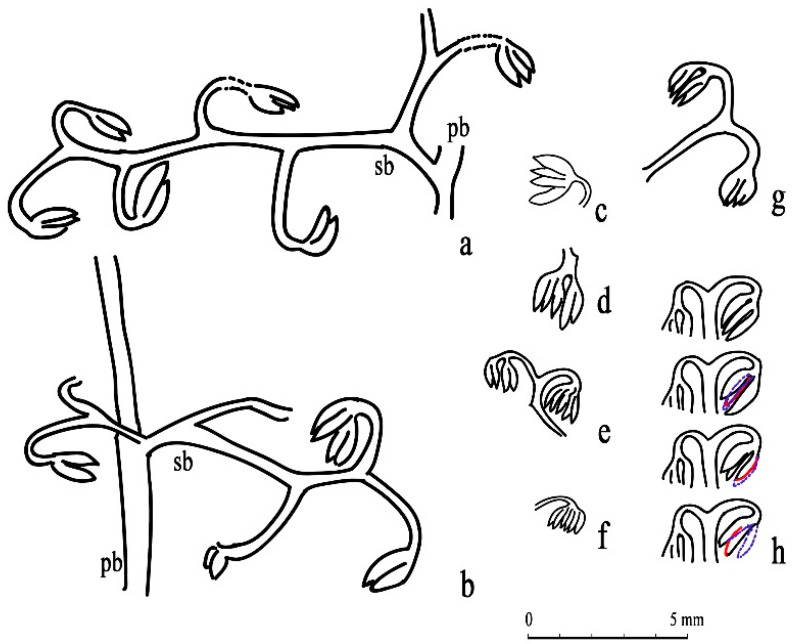
Morphology of *Xinhangia spina* gen. et sp. nov. from Guangde City, Anhui Province, China. (**a**–**h**) Line drawing of part of fertile branches and fertile organs in Figure 5 showing terminal sporangial pairs. (**a**–**g**) correspond to Figure 5D,G,A,C,I,K, respectively, and (**h**) corresponds to the serial dégagement of Figure 5T–W, where blue dotted lines and red lines indicate removed and newly exposed parts, respectively. pb, primary branch; sb, secondary branch.

**Figure 7:**
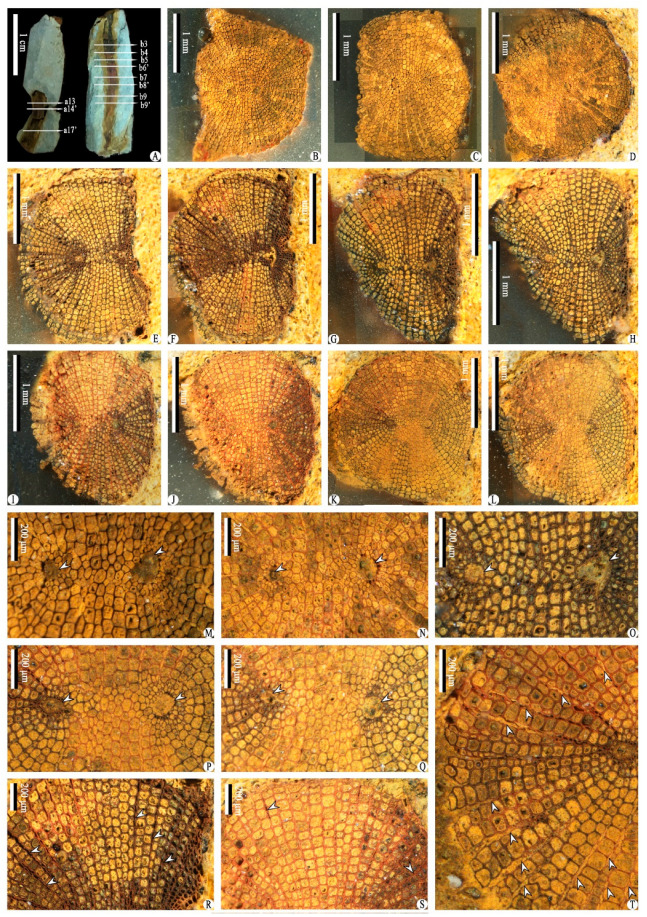
Anatomy of *Xinhangia spina* gen. et sp. nov. from Guangde City, Anhui Province, China. (**A**) Two axes before transverse sectioning. (**B**–**D**) Serial sections of the left axis in (**A**), showing clepsydroid-shaped primary xylem surrounded by secondary xylem (a13, a14′, a17′). (**E**–**L**) Serial sections of the right axis in (**A**), showing clepsydroid-shaped primary xylem surrounded by secondary xylem (b3, b4, b5, b6′, b7, b8′, b9, b9′). (**M**–**Q**) Close-up views of primary xylem of (**B**,**D**,**G**,**K**,**L**), respectively, showing two prominent protoxylem poles (arrows) and primary xylem tracheids. (**R**–**T**) Close-up views of secondary xylem of (**F**,**J**,**L**), respectively, showing radial files of secondary xylem tracheids and rays. Arrows indicate rays.

**Figure 8:**
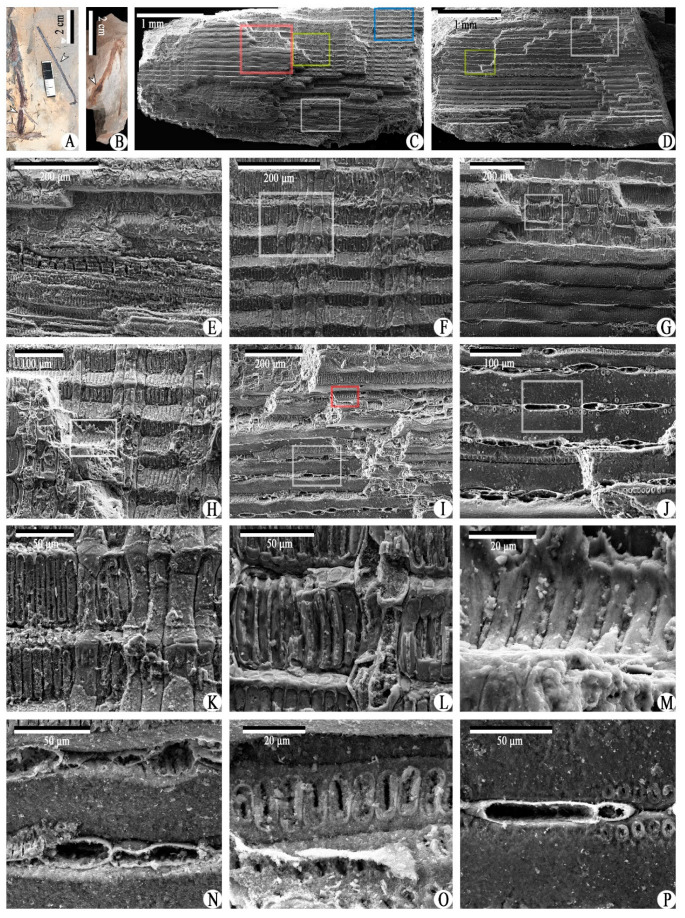
Anatomy of *Xinhangia spina* gen. et sp. nov. from Guangde City, Anhui Province, China. (**A**) Specimen from which axis in **C** was obtained (left arrow), and right arrow indicating a spiny axis. (**B**) Specimen from which axis in (**D**) was obtained (arrow). (**C**,**D**) Overview of SEM of two axes from (**A**,**B**) representing longitudinal and tangential sections, respectively. (**E**–**J**) Enlargement of the white, blue, red and yellow rectangle areas in (**C**) and the white and yellow rectangle areas in (**D**), respectively, showing xylem tracheids and ray cells. Rectangles in (**F**–**J**) are enlarged in (**K**–**P**). (**K,L**) Enlargement of rectangle areas in (**F**,**G**), showing scalariform thickenings and ray cell walls. (**M**) Enlargement of rectangle area in (**H**), showing scalariform thickenings. (**N**) Enlargement of the white rectangle area in (**I**), showing transverse view of ray cells. (**O**) Enlargement of the red rectangle area in (**I**), showing bordered pits. (**P**) Enlargement of rectangle area in (**J**), showing transverse view of ray cells and bordered pits.

- Class and Order: *Incertae sedis*
- Genus: *Xinhangia* Yang et Wang gen. nov.
- Type species: *Xinhangia spina* Yang et Wang gen. et sp. nov.
- Generic diagnosis: Main axes dichotomous. Primary and secondary branches borne alternately and sometimes in triseriate pattern. Vegetative or fertile ultimate appendages arranged alternately on secondary branch. Vegetative ultimate appendages with recurved tips usually dichotomizing 1–2 times. Fertile ultimate appendages usually dichotomizing 1–2 times to terminate in elongated and paired sporangia. Primary xylem mesarch, clepsydroid-shaped with two protoxylem poles, and surrounded by secondary xylem. Secondary xylem rays uniseriate. Tracheid wall with scalariform thickenings or circular to elliptical bordered pits.
- Etymology: The generic name derived from Xinhang Town, indicating the locality where the new plant was collected.
- *Xinhangia spina* Yang et Wang gen. et sp. nov. (Figure 1A–K, Figure 2a,b, Figure 3A–I, Figure 4A–M, Figure 5A–M, Figure 6a–h, Figure 7A–T and Figure 8A–P).
- Specific diagnosis: As in the generic diagnosis. Main axes 1.8–6.0 mm wide and up to 111 mm long, with primary branches arranged at 45–70°. Primary branches 0.7–3.0 mm wide and up to 65 mm long; secondary branches 0.3–1.0 mm wide and up to 50 mm long; tertiary branches 0.2–0.4 mm wide and up to 7 mm long. Sometimes a dichotomous aphlebia inserted at the base of primary and secondary branches. Spines on main axes and primary axes, 1–3 mm long. Basal axes within fertile ultimate appendages 0.2–0.4 mm wide. Sporangia 0.3–0.5 mm wide and 0.9–1.5 mm long. In main axes, xylem column 2 mm in diameter. Primary xylem 220–360 μm by 720–870 μm in transverse section. Tracheids in protoxylem, metaxylem and secondary xylem 10–25 μm, 30–51 μm, 32–73 μm in diameter, respectively.
- Etymology: The specific name referring to the occurrence of spines.

## 4. Description

The description of *Xinhangia* involves its morphology (Figure 1, Figure 2, Figure 3, Figure 4, Figure 5 and Figure 6) and anatomy (Figure 7 and Figure 8). The measurements of the morphology and anatomy are given in Table 1.

### 4.1. Main Axes and Primary Branches

The main axes or stems have an upright habit and are covered by dense spines of 1–3 mm in length (Figure 1A–F and Figure 2a). One main axis is dichotomous, up to 6 mm in diameter and 11 cm in length (Figure 1A,B). The main axes sometimes present a slightly zigzag shape, bending at the position where the primary branches occur (Figure 1F). No roots are found on the main axes.

The primary branches are straight in most cases (Figure 1F–H and Figure 3B,D) and slightly zigzag in some instances (Figure 3E and Figure 4A). They are arranged on the main axes in alternate (Figure 1E) or triseriate (Figure 1F and Figure 2a) branching patterns at 45–70°. The paired primary branches (Figure 1F, arrows 2, 3) and a single primary branch (Figure 1F, arrow 6) constitute a triseriate pattern. A presumed aphlebia is dichotomous and inserted at the base of a primary branch (Figure 1J). The primary branches are 0.7–3.0 mm in diameter and up to 6.5 cm long. Compared to those on the main axes, the spines on the primary branches are sparse and short. No ultimate appendages are visible on the primary branches.

### 4.2. Vegetative Secondary Branches and Vegetative Ultimate Appendages

The secondary branches are alternately arranged on the primary ones at 50–90° (Figure 1F and Figure 3D,F). They lack spines and measure 0.4–0.7 mm in diameter and up to 5 cm long. A single dichotomous aphlebia is located at the base of some of the secondary branches (Figure 3B, arrow 2; Figure 3C, arrow 2; Figure 3G, arrow 1). The aphlebiae are similar in shape to the vegetative ultimate appendages but appear a little larger. The upper portion of a secondary branch is preserved, tapered and appears distally recurved (Figure 3I).

The vegetative ultimate appendages occur alternately on the secondary branches and bear recurved tips (Figure 3). Most of these appendages dichotomize once to form a ‘Y’ shape (Figure 3A, arrows 1, 3; Figure 3B, arrow 1; Figure 3E, arrows 1, 2; Figure 3G, arrows 2, 3, 5). Some appendages dichotomize twice (Figure 3A, arrow 2; Figure 3G, arrow 4) or thrice (Figure 3G, arrows 6–8), or not (Figure 3H,I).

### 4.3. Fertile Branches

Most of the fertile secondary branches occur alternately on the primary branches at 50–90° (Figure 1H and Figure 4A). However, as exemplified by one specimen (Figure 1G,H,K and Figure 2b), three fertile secondary branches alternate on the lower part of the primary branch; a pair of fertile secondary branches and a single branch occur oppositely or sub-oppositely on the upper part of the primary branch. In this example, these fertile secondary branches appear to form a triseriate branching pattern. The fertile secondary branches (e.g., Figure 4E,I) are quite similar to the vegetative ones (e.g., Figure 3A,B); their only difference lies in the separate occurrence of fertile and vegetative ultimate appendages. A complete secondary branch shows alternate fertile ultimate appendages (fertile organs) and terminates in one fertile organ (Figure 5D and Figure 6a). No aphlebiae are found inserted at the base of the secondary branches.

Tertiary branches were discovered only in one specimen (Figure 1H). They are alternately arranged and bear fertile organs (Figure 2b, tb), resembling fertile secondary branches (e.g., Figure 4G–I) in shape and size.

### 4.4. Fertile Organs

Fertile organs are inserted mostly on the secondary branches (Figure 4) and rarely on the tertiary ones (Figure 2b). A fertile organ consists of two parts, i.e., the terminal sporangia and a basal axis. The sporangia with pointed tips are elongate in shape and borne in pairs (Figure 5 and Figure 6). They range 0.3–0.5 mm wide and 0.9–1.5 mm long. The smooth basal axes within the fertile organs measure 0.2–0.4 mm in diameter and usually dichotomize but sometimes do not. In the distal area of the secondary branches, such axes lack dichotomy and terminate in one pair of sporangia (Figure 5E,N). In most of the examples, the basal axes dichotomize once or twice to produce two or four pairs of terminal sporangia (Figure 5H,O,P,T and Figure 6g). Occasionally, the basal axes dichotomize thrice to form possibly eight pairs of sporangia (Figure 5I,J and Figure 6e). As to one fertile organ with twice dichotomizing, the serial dégagement shows two pairs of terminal sporangia in three dimensions (Figure 5T–W and Figure 6h).

### 4.5. Anatomy

Two limonitized axes containing only the xylem, ca. 2 mm in diameter, were embedded and transversely sectioned into 18 and 15 slices, respectively (Figure 7A). Of these, 11 slices are selected to show the relatively complete structure (Figure 7B–L). The stele has a primary xylem surrounded by a radial secondary xylem. Though these two axes were preserved as isolates without an organic connection with the morphological parts, we believe that they belong to *Xinhangia,* with a high probability, because of the close relationship of the preservation and correspondence between the two-poled pattern in the anatomy and the alternate branching pattern in the morphology. For the limonitized axis observed by SEM (Figure 8A, left arrow), a spiny axis was preserved beside it (Figure 8A, right arrow), indicating the close relationship among the axes in Figure 8A. Considering the tissues outside the xylem and the diameter of the main axes and branches, it is assumed that these limonitized axes represent the main axes. 

The primary xylem, 720–870 μm by 220–360 μm in the transverse section, is mesarch in maturation and has a clepsydroid shape in the cross-section (Figure 7M–Q). At each end of the primary xylem, there is a prominent protoxylem pole (Figure 7M–Q, arrows), which may have been originally filled with parenchymal cells. The smallest tracheids surrounding the protoxylem poles indicate the protoxylem and measure 10–25 μm in diameter. The bigger tracheids between and surrounding the protoxylem represent metaxylem and 30–51 μm in diameter.

The tracheids of the secondary xylem are arranged in radial files (Figure 7R–T) and are 32–73 μm in diameter. In the transverse sections, the rays can be recognized between the rows of the secondary xylem tracheids (Figure 7R–T, arrows). With SEM, the uniseriate rays appear to occur between every row of the secondary xylem tracheids (Figure 8D, J,N,P). A single ray is one to six cells in height (Figure 8C,F,H,K). The secondary wall of the secondary xylem tracheids may possess scalariform thickenings (Figure 8L,M). In the tangential section (Figure 8D), circular to elliptical bordered pits are visible in the walls of the secondary xylem tracheids (Figure 8J,O,P).

## 5. Comparisons

Table 2 indicates comparisons among *Xinhangia*, other fern-like plants and related groups. The comparisons involve the vegetative branching pattern, basal aphlebiae, fertile organs (fertile ultimate appendages) and stelar architecture.

### 5.1. Iridopteridales, Pseudosporochnales and Nonpseudosporochnaleans

Middle Devonian–Early Carboniferous Iridopteridales and Pseudosporochnales are assigned to the Cladoxylopsida [9]. Alternatively, the Pseudosporochnales and nonpseudosporochnaleans, as an informal group, are placed in this class [10]. The recent phylogenetic analysis regards that the cladoxylopsids *sensu lato* refer to the iridopteridaleans and cladoxylopsids *sensu stricto*, which include the pseudosporochnaleans and nonpseudopsorochnaleans [23]. In general, the iridopteridaleans refer to plants such as *Anapaulia* [24], *Arachnoxylon* [25], *Asteropteris* [26], *Compsocradus* [27], *Ibyka* [28], *Iridopteris* [29], *Keraphyton* [30] and, possibly, *Metacladophyton* [12,31], the pseudosporochnaleans to *Calamophyton* [32], *Lorophyton* [33], *Pseudosporochnus* [11] and *Wattieza* [34], and the nonpseudosporochnaleans to the taxa such as *Cladoxylon* [35], *Panxia* [36,37], *Pietzschia* [1,38], *Polyxylon* [10] and, possibly, *Denglongia* [13,14]. 

*Xinhangia* and most cladoxylopsids *s.l.* share similar fertile organs (three-dimensional dichotomous branches, terminated by elongate and paired sporangia) and mesarch primary xylem. However, *Xinhangia* is characterized by a triseriate branching pattern and clepsydroid stele, which are absent in the cladoxylopsids *s.l*. 

Whorled organs (the branches and ultimate appendages) and actinostele (a protostele with the primary xylem ribs arranged radially and more or less connected in the stelar center) occur in all the iridopteridaleans but not in *Xinhangia*. The digitate branching and dissected stele characterizing the pseudosporochnaleans are absent in *Xinhangia*. Furthermore, the iridopteridaleans and pseudosporochnaleans usually have no secondary xylem. Dissected stele typifies (most) nonpseudosporochnaleans but is not recorded in *Xinhangia*.

Among the nonpseudosporochnaleans, *Panxia* [36,37] is known for its morphology. As with *Xinhangia*, *Panxia* has alternate branches and simple vegetative ultimate appendages with distal recurving. Nevertheless, its discoidal sporangia with stalks are borne laterally and closely. *Metacladophyton* [12,31] is treated as a nonpseudosporochnalean [23]. It morphologically differs from *Xinhangia* mainly in the whorled and decussate branches.

*Denglongia* [13,14] is placed in the cladoxylopsids *s.s.*, but is not allied with the nonpseudosporochnaleans or nonpseudosporochnaleans [23]. It shows clear differences with *Xinhangia* in the whorled branches, complex and unique fertile organs with alternate segments and actinostele.

### 5.2. Rhacophyton with (Possibly) Related Plants

Late Devonian (Famennian) *Rhacophyton* is characterized by quadriseriate (alternate pairs of) branches with basal aphlebia and a clepsydroid-shaped primary xylem surrounded by a secondary xylem [15,16,17,39]. *Rhacophyton* is assigned to the Rhacophytales, relating to plants such as *Ellesmeris*, *Eocladoxylon*, *Melvillipteris* and *Protopteridophyton* [2,3,6], which have quadriseriate branching and/or the clepsydroid stele preserved. 

As in *Xinghangia*, *Eocladoxylon* [19], *Melvillipteris* [6] and *Protopteridophyton* [20] show elongate and sometimes paired sporangia terminating in three-dimensionally dichotomous axes; *Ellesmeris* [18], *Eocladoxylon*, *Melvillipteris* and *Rhacophyton* possess basal aphlebiae; *Ellesmeris*, *Eocladoxylon* and *Rhacophyton* demonstrate clepsydroid xylem. However, all of these plants lack triseriate branching and some present quadriseriate branching; *Rhacophyton* has fertile organs paired at the branch base, and such arrangement occurs sometimes in *Eocladoxylon*; the fertile organs of *Rhacophyton* are very complex and unique, with pinnate segments, and those of *Protopteridophyton* are quadriseriate in arrangement and also complex.

### 5.3. Stauropteridales

Late Devonian–Carboniferous Stauropteridales [2] is characterized by quadriseriate branching, usually single sporangium terminating branches and actinostele usually with four primary xylem ribs and without secondary xylem. Such morphological and anatomical features are lacking in *Xinhangia*. Among the members, *Multifurcatus* has trichotomous branches, basal aphlebiae and a single sporangium at the node of the branches [39]. Its trichotomous branches appear to form alternate pairs. In contrast, *Xinghangia* possesses triseriate branches and terminal sporangia in pairs. 

### 5.4. Shougangia

Late Devonian (Fammenian) *Shougangia* is unknown for affinity at the class level. As a derived fern-like plant, it possesses helical branches, laminate vegetative leaves, very complex fertile organs, which terminate in branches with pinnules and dichotomize up to 10 times, and dissected stele [4,5]. These traits show clear differences from *Xinhangia*, although both genera have terminal and elongate sporangia in pairs.

### 5.5. Aneurophytales

Middle to Late Devonian (Late Eifelian to Frasnian) Aneurophytales is considered the most primitive progymnosperm [2,22], which is characterized by: (1) three-dimensional branching systems with laterals helically or decussately arranged, (2) a primary xylem stele consisting of three or more ribs with protoxylem strands occurring near the tips and along the midplanes of the ribs, (3) elongate sporangia terminally inserted on the ultimate pinnate fertile appendages [2]. *Xinhangia* exhibits an alternate and sometimes a triseriate branching pattern, which differs from the Aneurophytales. The clepsydroid-shaped primary xylem and protoxylem strands occurring around the two poles in *Xinhangia* distinguish it from the order. Furthermore, the Aneurophytales shows more complicated style in their fertile appendages, while the fertile organs of *Xinhangia* are simpler, by contrast.

## 6. Discussion

*Xinhangia* and most fern-like plants share fertile organs with three-dimensionally dichotomous axes terminated by elongate and paired sporangia and mesarch primary xylem with a permanent protoxylem (strand near the periphery of the xylem). Nevertheless, this taxon cannot be placed in the Iridopteridales, with whorled organs and actinostele, or in the Pseudosporochnales, with digitate branching and dissected stele, or in the nonpseudosporochnaleans with dissected stele. It cannot be assigned to the Rhacophytales and Stauropteridales, which typically have a quadriseriate branching pattern. It also cannot be classified into Aneurophytales, for their helical or decussate branching pattern, multiple ribs in the stele and the complex fertile appendages. Thus, *Xinhangia* is now treated as a fern-like genus of an uncertain affinity at the class or order level.

*Rhacophytonceratangium* and *Ellesmeris sphenopteroides* possess both quadriseriate branching and clepsydroid xylem. Specifically, in *R. ceratangium*, the common bases of each pair of primary branches are alternately attached to the main axis or stem [17,39]; in *E. sphenopteroides*, the common bases of the paired primary branches have the same arrangement, and the secondary branches are unpaired and borne alternately or suboppositely [18]. Either the main axes or primary branches in both plants contain a clepsydroid stele. By contrast, in *R. zygopteroides*, the primary branches are helical on the main axis (probably) containing an actinostele [15]. Alternate branching and clepsydroid stele are also seen in *Eocladoxylon* [19] and now, in *Xinhangia*. As to the triseriate primary or secondary branches of *Xinhangia*, the unpaired branches and the common base of the paired branches are alternately attached. Therefore, in these four plants, the common bases of the paired branches and/or unpaired branches occur in a single plane, correlating to the clepsydroid stele with the primary xylem bearing two peripheral protoxylem strands. This suggests that the clepsydroid stele may not be highly stressed in discussing relationships among plants such as rhacophytaleans.

The co-evolution of the vegetative and fertile organs has been suggested for the fern-like plants [4]. The derivation of the laminate leaves from planate ultimate appendages is accompanied by the increase in complexity of the fertile organs (number of internal divisions and terminal sporangia). Of *Xinhangia* as a plesiomorphic taxon, the vegetative ultimate appendages, with the distal recurving and rare divisions, are simple and far from planation. Accordingly, the fertile organs divide usually only 1–2 times to produce few terminal sporangia. The secondary xylem provides mechanical support and, fundamentally, water conductance for fern-like plants with planate ultimate appendages or laminate leaves, e.g., *Rhacophyton* and *Shougangia* [3,5]. As to *Xinhangia* lacking leaf-like organs or leaves, the secondary xylem appears necessary to perform a supporting function. Of course, the explanation of such a function in *Xinhangia* may also depend on the growth habit and understanding of its habitat (under study).

## 7. Conclusions

*Xinhangia spina* gen. et sp. nov. is known from both its morphology and anatomy. It is characterized by primary and secondary branches borne in an alternate and sometimes a triseriate pattern, vegetative and fertile ultimate appendages with rare divisions and a clepsydroid-shaped primary xylem surrounded by secondary xylem. By comparison with other fern-like plants, *Xinhangia* represents a new and primitive taxon with uncertain affinity. Based on stelar architecture in relation to branching pattern, the anatomical features of some fern-like plants are reconsidered.

## Figures and Tables

**Table 1:** Measurements of morphology and anatomy of *Xinhangia spina*.

Morphological Organs	Length (mm)	Diameter (mm)
main axes	up to 111	1.8–6.0
primary branches	up to 65	0.7–3.0
secondary branches	up to 50	0.3–1.0
tertiary branches	up to 7	0.2–0.4
axes within fertile organ	-	0.2–0.4
sporangia	0.9–1.5	0.3–0.5
**Anatomical Structures**		
primary xylem	720–870	220–360
protoxylem tracheids	-	10–25
metaxylem tracheids	-	30–51
secondary xylem tracheids	-	32–73

**Table 2:** Comparisons of *Xinhangia* with fern-like and related plants *.

Taxon (Reference)	Vegetative Branch			Basal Aphlebia	Fertile Organ		Anatomy	
	Primary	Secondary	Tertiary		Arrangement Branching Pattern	Sporangia	Stelar Structure	Secondary Xylem
*Xinhangia*	alternate triseriate	alternate triseriate	alternate	present	alternate dichotomous 1–2 times	terminal, paired, elongate	clepsydroid	present
Iridopteridales [9,10]	whorled			absent	whorled 3-D dichotomous	terminal, paired, elongate	actinostele	usually absent
Pseudosporochnales [9,10,11]	digitate			absent	usually irregular mostly 3-D dichotomous	terminal, paired, elongate	dissected	usually absent
*Metacladophyton* [12]	whorled	decussate opposite	absent	absent	alternate 3-D dichotomous about 6 times	terminal, single, elongate	protostele dissected	present
*Denglongia* [13,14]	whorled			absent	subopposite 3-D dichotomous many times	along inner side of alternate segments, elongate	actinostele	absent
*Rhacophyton* [15,16,17]	quadriseriate helical	quadriseriate alternate	alternate	present	paired at secondary branch base 3-D dichotomous many times	terminal on pinnate segments, elongate	actinostele clepsydroid	present
*Ellesmeris* [18]	quadriseriate alternate	alternate subopposite	absent	present	unknown		clepsydroid	absent
*Eocladoxylon* [19]	alternate			present	paired at branch base or alternate on branch 3-D dichotomous 2–3 times	terminal, paired, elongate	clepsydroid	absent
*Melvillipteris* [6]	quadriseriate	alternate	absent	present	alternate 3-D dichotomous up to 5 times terminal, elongate	unknown	unknown	unknown
*Protopteridophyton* [20]	helical in two ranks	quadriseriate	quadriseriate	absent	quadriseriate 3-D dichotomous about 6 times	terminal, paired, elongate	? actinostele	absent
*Stauropteridales* [2,21]	quadriseriate, trichotomous			present	few (single or paired) sporangia terminal on aphlebia-like branch or on branch node		actinostele	absent
*Shougangia* [4]	helical	irregularly helical	alternate (sub)–opposite	absent	terminal on tertiary branch with pinnules 3-D dichotomous up to 10 times		terminal, paired, elongate	present
Aneurophytales [2,22]	helical or decussate			absent	terminal on pinnate divisions of a dichotomous branching system		actinostele	present

* modified from Table 2 in [4]; 3-D, three dimensionally; ? actinostele, possible actinostele.

## Data Availability

The specimens are deposited in the Department of Geology, Peking University, Beijing, China.
